# Establishment and identification of a rabbit model of peritoneal carcinomatosis from gastric cancer

**DOI:** 10.1186/1471-2407-10-124

**Published:** 2010-04-01

**Authors:** Lie-Jun Mei, Xiao-Jun Yang, Li Tang, Alaa Hammed al-shammaa Hassan, Yutaka Yonemura, Yan Li

**Affiliations:** 1 Department of Oncology, Zhongnan Hospital of Wuhan University & Hubei Key Laboratory of Tumor Biological Behaviors, Wuhan, 430071, China; 2 NPO Organization to Support Peritoneal Dissemination Treatment, Osaka, Japan

## Abstract

**Background:**

Gastric cancer peritoneal carcinomatosis is a common clinical problem, but there are no suitable large animal models to study this problem. This study was to establish a stable rabbit peritoneal carcinomatosis model of gastric cancer using VX2 tumor, and analyze the clinico-pathological features.

**Methods:**

VX2 tumor was implanted into 36 New Zealand rabbits by 3 methods: laparotomic orthotopic injection of cancer cells into the submucosal layer of the stomach (Group A), laparotomic implantation of tumor tissue into the greater omentum immediately beneath the gastric antrum (Group B), and percutaneous injection of tumor cells directly into the peritoneal cavity (Group C), 12 rabbits in each group. The animals were closely observed and detailed clinico-pathological studies were conducted.

**Results:**

The success rates of peritoneal carcinomatosis formation were 100% (12/12), 91.7% (11/12) and 58.3% (7/12), respectively, for Groups A, B and C (P = 0.019, A versus C; P = 0.077, B versus C; P = 0.500, A versus B, Fisher's exact test). Two weeks after submucosal cancer cells injection in Group A, ulcerative gastric cancer with peritoneal carcinomatosis showed typical VX2 tumor pathology, with widespread intraperitoneal metastatic nodules, bloody ascites and perspicuous pulmonary metastases. The clinico-pathological progression pattern was very similar to patients of advanced gastric cancer with peritoneal carcinomatosis. Groups B and C showed similar pattern of cancer progression, but less aggressive.

**Conclusions:**

First large animal model of peritoneal carcinomatosis from gastric cancer has been established by laparotomic orthotopic injection of VX2 cancer cells into the submucosal layer of the stomach, providing a more suitable model for surgical interventional studies. The clinico-pathological features of this model resemble human peritoneal carcinomatosis.

## Background

The loco-regional progression of gastrointestinal and gynecological cancers frequently results in peritoneal carcinomatosis (PC), which is characterized by the presence of tumor nodules of various size, number and distribution on the peritoneal surface, with very poor prognosis and a median survival of less than 6 months [1,2]. Current treatments for such PC are systemic chemotherapy, best support care and palliative therapy, with no hope of cure. In order to tackle this problem, a new treatment modality called cytoreductive surgery (CRS) plus hyperthermic intraperitoneal chemotherapy (HIPEC) has been developed over the past two decades, taking advantages of surgery to reduce visible tumor burden, and regional hyperthermic chemotherapy to eradicate micrometastases [3-6].

While clinical studies have made progresses, some experimental animal PC models have also been developed, including mice models and rat models to evaluate the efficacy and adverse effects of experimental HIPEC protocols [7-10]. Although such small animal models are useful in experimental studies, it is technically difficult to perform operations on small animals because of the little body size and delicate hemodynamic conditions. Large animal such as pig has also been used to test the pharmacokinetics of HIPEC, but the animals used were healthy pigs rather than pigs with PC [11]. Therefore, a large animal PC model more suitable for surgical interventional studies is desirable. Here we report on a rabbit PC model from gastric cancer, with clinico-pathological features mimic patients with advanced gastric cancer.

## Methods

### Animals

Thirty six New Zealand white rabbits, 18 males and 18 females, body weight between 2.5~3.0 kg, were obtained from Animal Biosafety Level 3 Laboratory at the Animal Experimental Center of Wuhan University (Animal Study Certificate SCXK 2003-0004). The animals were individually housed and allowed free access to standard laboratory food and water as well as 12 h of light and dark cycle per day. The animal study protocol was approved by the Animal Welfare Committee of the Center.

### Tumor strain and tumor cell preparation

Rabbit VX2 carcinoma was used to establish gastric cancer with PC in this study. The VX2 tumor is a transplantable rabbit squamous cell carcinoma, characterized by rapid tumor growth and early metastasis, established from a virus-induced papilloma by Rous and coworkers [12]. The tumor was maintained by successive *in vivo *transplantation into the hind leg of 2 carrier rabbits used for every passage.

When the VX2 tumor grew to about 1 cm in diameter on the carrier rabbit, the animal was anesthetized by ear vein injection of 3% pentobarbital sodium (30 mg/kg). After skin preparation and disinfection, the tumor was excised from the carrier rabbit and placed in icy cold 0.9% sodium chloride solution. Tumor tissue was minced into approximately 1.0~2.0 mm^3 ^fragments and suspended in 2 mL of normal saline, then drawn into a 2 mL injector. Other tumor tissues about 3.0~5.0 mm^3 ^were placed into the homogenizer embedded in ice bath, to which 3 mL of icy cold normal saline was added, and the tumor cells suspension was made, with the tumor cells concentration adjusted to 5×10^10 ^vial cells/L.

### Construction of gastric cancer with PC

**Figure 1:**
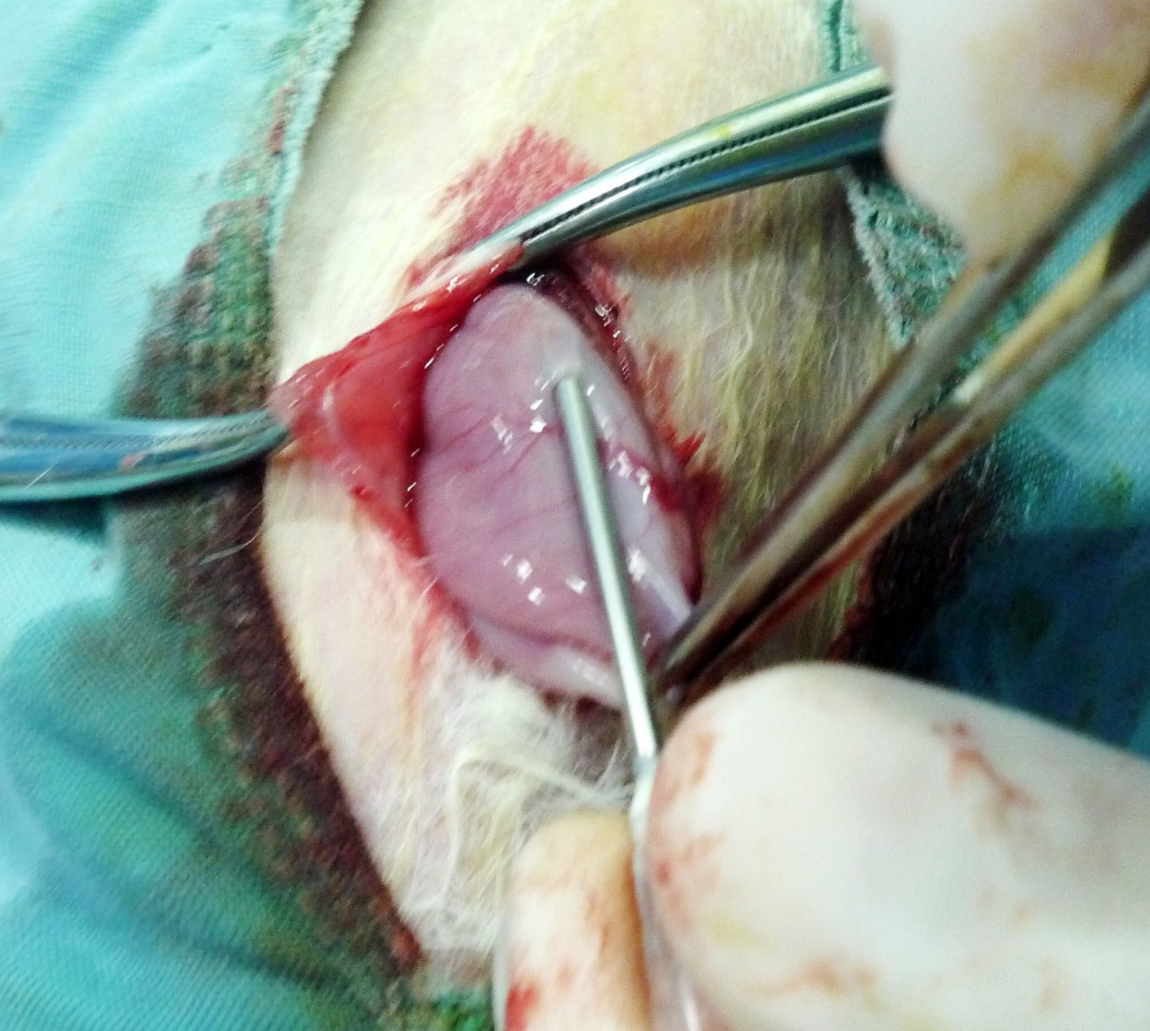
**Construction of rabbit peritoneal carcinomatosis model from gastric cancer**. When the rabbit stomach was exposed under general anesthesia, a 16 G needle was inserted through the serosal and muscle layers into the submucosal layer of the stomach, and 0.1 mL of tumor cells (5×10^10^ vial cells/L) were injected, and injection site was pressed for 1 min to keep the tumor cells in place (See the text for detailed description).

All rabbits had overnight fasting before experiment, but water was given *ad libitum*. After randomization, the animals were anesthetized by ear vein injection of 3% pentobarbital sodium (30 mg/kg). The abdominal skin was cleaned and disinfected. Three approaches were adopted to construct rabbit models of PC, 12 animals for each group. Group A of submucosal tumor cell inoculation: A midline incision of 3 cm long was made beginning 2 cm below the xyphoid and the upper abdomen was open. The stomach was exposed, 0.1 mL of tumor cells (5×10^10 ^vial cells/L) was injected into the submucosal layer of the stomach (Figure 1), through the serosal layer and the muscle layer, the injection site was pressed for 1 min to keep the injected tumor cells in place, and the abdomen was closed with a double layer 3-O vicryl interrupted suture. Group B of tumor tissue implantation: The incision was the same as in Group A. When the stomach was exposed, a small piece of fresh tumor tissue about 1.0 mm^3 ^was implanted into the greater omentum immediately beneath the gastric antrum, and the wound was closed. Group C of percutaneous injection of tumor cells: After skin preparation, 0.1 mL of tumor cells (5×10^10 ^vial cells/L) was directly injected into the upper abdominal cavity, and the injection site was pressed for 1 min. After tumor inoculation, penicillin G at the dose of 100,000 IU/d was intramuscularly injected to each animal for 3 days. All the animals were give intravenous fluid rehydration with 100 mL of 0.9% normal saline solution.

### Animal observation and pathological studies

After operation, daily observation was made on each rabbit to check food intake, activities, and any abnormalities such as diarrhea and dehydration. The body weight was measured every 3 days and the natural history of the disease progression was recorded. In order to obtain a detailed description of the progressive development of gastric cancer PC, euthanasia was performed on 3 rabbits in Group A at the end of weeks 1, 2, 3 and 4, by overdose injection of 3% pentobarbital sodium through the ear vein. For animals in Groups B and C, euthanasia was also performed when the animals showed obvious signs of distress and waste, in keeping with UKCCCR guidelines for the welfare of animals in experimental neoplasia [13]. Post mortem pathological examinations included gross pathology such as tumor size and distributions; local tumor features of gastric cancer including ulcer formation, obstruction and perforation; special features of peritoneal carcinomatosis such as bloody ascites, discrete or confluent tumor nodules on the peritoneum, cancerous changes in the greater omentum and intestinal obstructions; metastases to major organs such as the liver, adrenal glands, pancreas and the lungs. All the suspected organ tissues were sampled for routine histopathology study with sections stained by hematoxylin and eosin (HE stain).

### Statistical analysis

The body weight and tumor weight were expressed as mean ± standard deviation (M ± SD). The intra-abdominal metastases and bloody ascites were recorded as ranges and medians. The SPSS statistical software package version 10.0 (SPSS Inc., Chicago Il, USA) was used for analysis, with two-sided test, and P < 0.05 was considered as statistically significant.

## Results

### Success of PC model construction

Of 36 animals used for PC model construction, the operation was successful in 33 rabbits and 3 animals died 3 days after operation. Of the 3 deaths, 1 rabbit in Group C died of diffused peritonitis on day 3 because the percutaneous injection was mistakenly into the small intestine; and 1 rabbit each in Groups B and C died of congestive heart failure on day 2 because of fast fluid rehydration. Another 3 rabbits in Group C did not develop PC for unknown reasons. The success rates were 100% (12/12) in Group A, 91.7% (11/12) in Group B and 58.3% (7/12) in Group C (P = 0.019, A versus C; P = 0.077, B versus C; P = 0.500, A versus B, Fisher's exact test).

### Tumor growth and its impact on the animal

After cancer cells injection into the gastric submucosa in Group A, the tumor demonstrated accelerated growth. The tumor sizes were (0.028 ± 0.012) cm^3^, (1.183 ± 0.148) cm^3 ^and (7.706 ± 1.629) cm^3^, respectively, at the end of weeks 1, 2, and 3. The animals showed progressive decrease in body weight, from (2.48 ± 0.21) kg before operation to (2.45 ± 0.21) kg on week 1, (2.24 ± 0.19) kg on week 2, (2.02 ± 0.18) kg on week 3 and (1.81 ± 0.1) kg on week 4. Along with body weight changes, the feeding and general health status of the animals deteriorated progressively.

### Growth characteristics of peritoneal carcinomatosis in animal model

**Figure 2:**
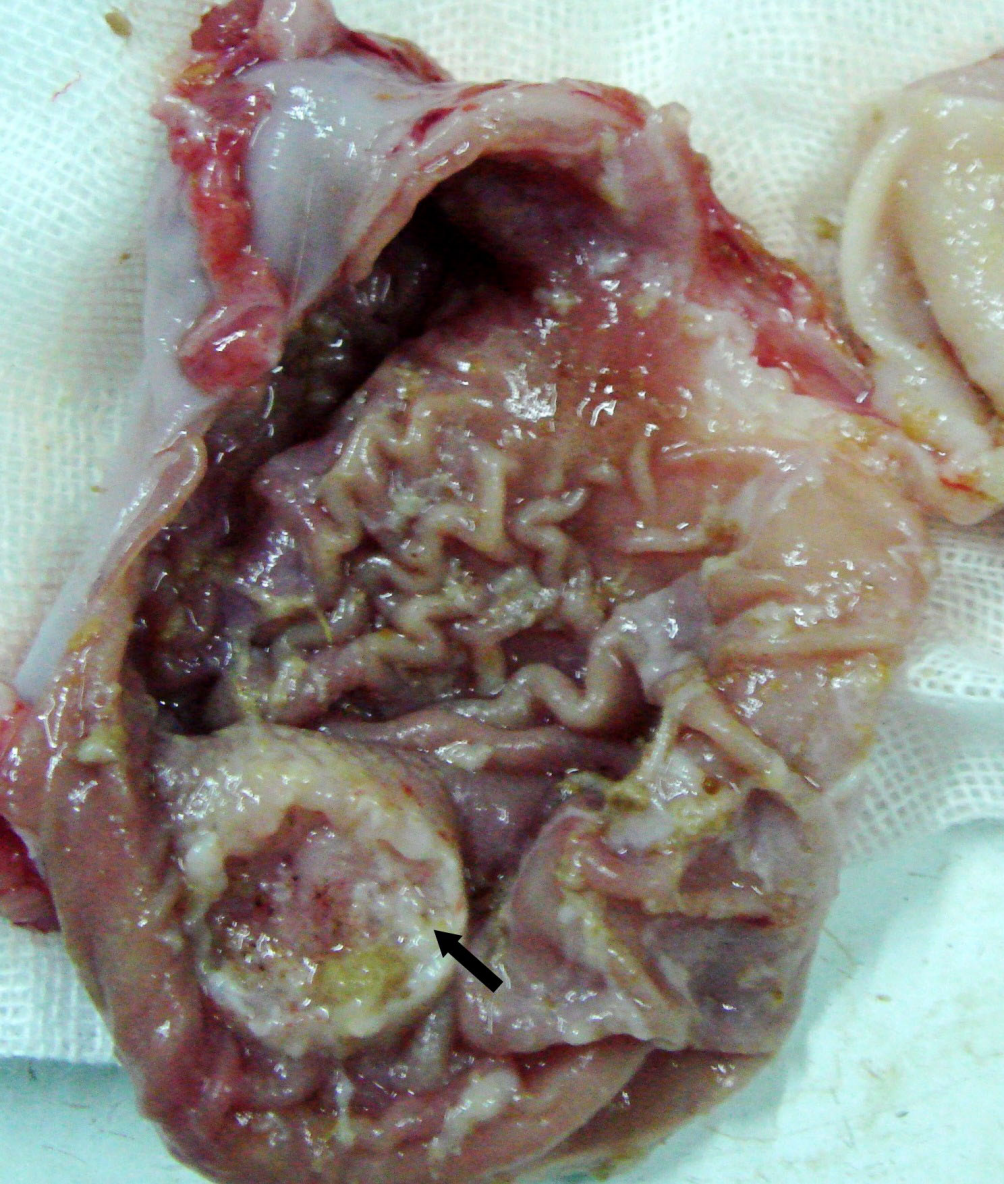
**Rabbit gastric cancer in group A**. Two weeks after submucosal inoculation of VX2 tumor cells, pathological study after animal euthanasia shows typical ulcerative gastric cancer (arrow) in New Zealand rabbit.

**Figure 3:**
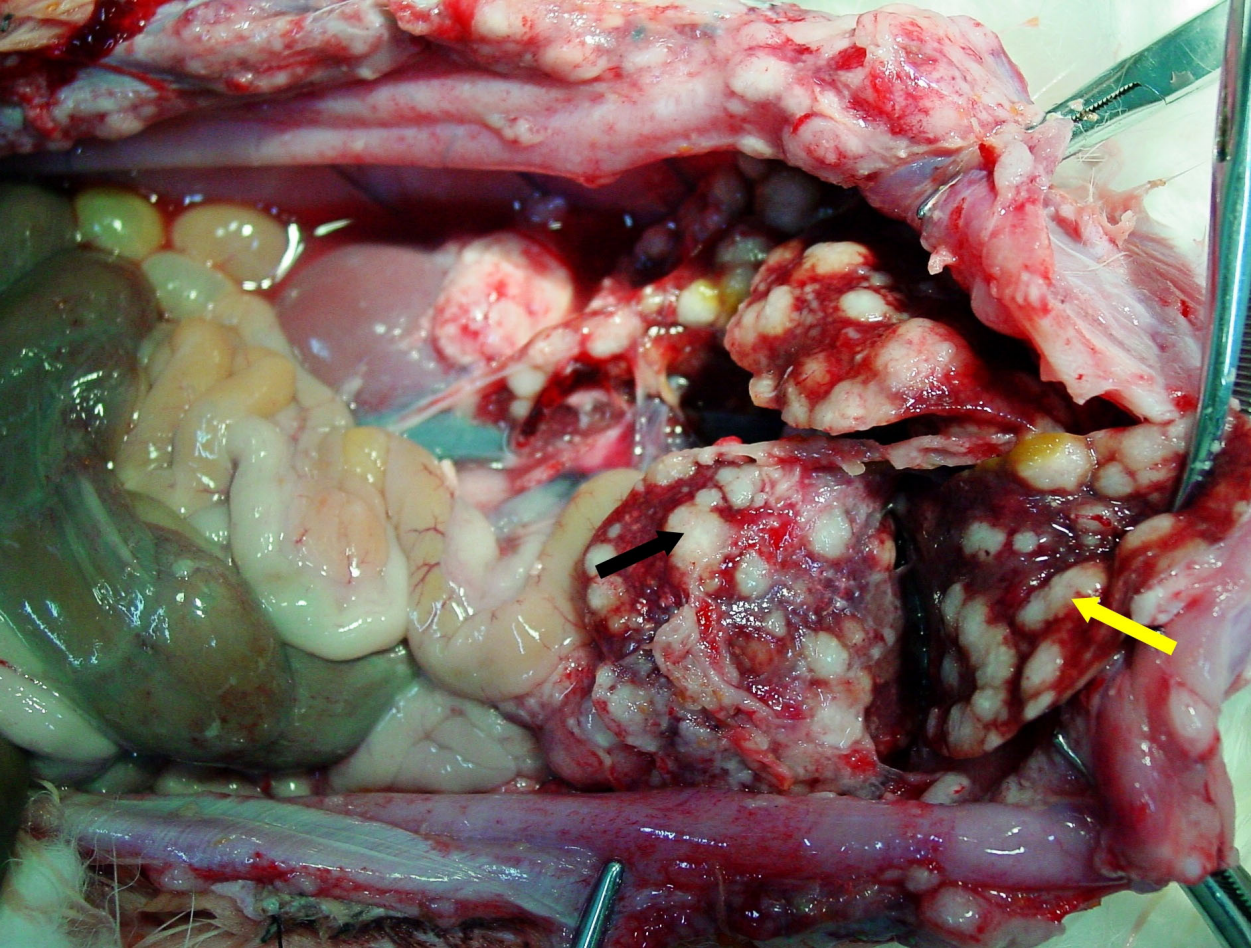
**Rabbit PC model of gastric origin from Group A**. Three weeks after VX2 carcinoma cells were injected into the submucosal layer of the stomach, confluent tumor mass on the greater omentum encased stomach wall (black arrow) and the liver (yellow arrow).

Tumor characteristics in Group A were carefully recorded. One week after submucosa injection of cancer cells, many small, hard and transparent nodules began to develop on the greater omentum and the antrum of the stomach. No ascites was observed. Two weeks later, nodules on the greater omentum began to merge into confluent masses without discernable demarcations with the stomach. The mass on the gastric wall grew bigger and protruded into the stomach cavity to form typical ulcerative cancer (Figure 2). There was about 5 mL of bloody ascites in the abdominal cavity. Three weeks later, many nodules began to form in the mesentery and the retroperitoneum. The confluent mass on the greater omentum began to invade the liver and encase the stomach (Figure 3). Often the stomach mass and the greater omentum mass merged into one big tumor block. Bloody ascites could reach as much as 100 mL. There was also some fluid accumulation in the pericardium. Many nodules were seen on the abdominal wall. No nodules were observed in the lungs. Four weeks later, numerous tumor nodules were observed in the lungs, the liver, adrenal glands, small intestine, transverse colon and the bladder. The abdominal wall was totally invaded by the tumor. Tumors in Groups B and C showed similar growth characteristics.

### Histopathological characteristics of VX2 rabbit PC

**Figure 4:**
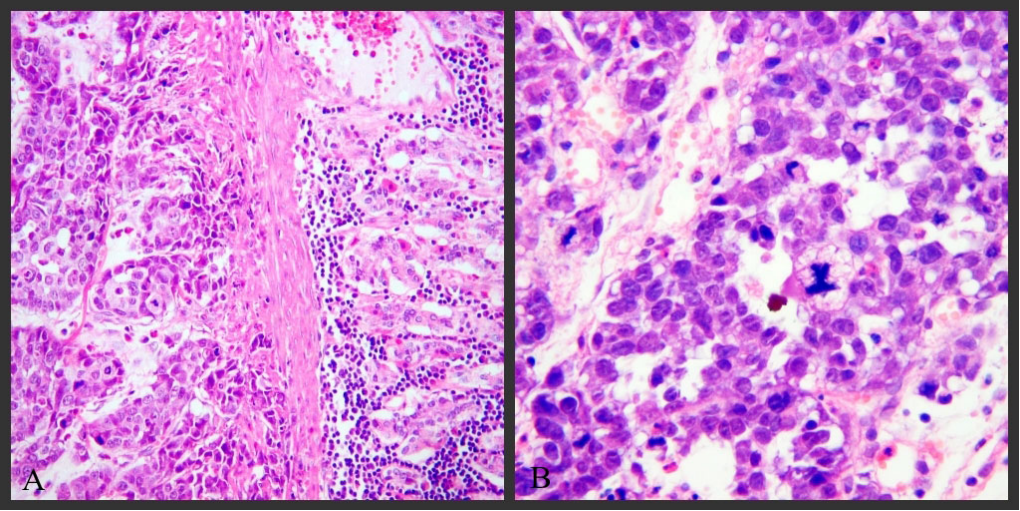
**Histopathology of rabbit model gastric cancer with PC, 2 weeks after VX2 carcinoma cells were injected into the submucosal layer of the stomach in Group A**. VX2 tumor cells invaded the whole stomach wall (4A, ×100, HE stain) and showed many pathological mitotic figures (4B, ×200, HE stain).

All investigated tumor specimens showed extensive invasive growth and tissue destruction. The tumors, on the greater curvature of the gastric antrum, penetrated the mucosal layer to form ulcers. Microscopic view could find cancer nests penetrating the whole stomach wall, with typical invasion into the muscle layer and the gastric glands (Figure 4A). The tumor cells are round, oval or atypical morphology with many pathological mitotic figures (Figure 4B). There were also conspicuous lymphocytes, plasma cells and other inflammatory cells infiltration.

Major features of this rabbit model of PC from gastric cancer could be summarized in Table 1.

**Table 1:** Tumor characteristics of three different inoculation approaches

	Group A	Group B	Group C
Technical feature	Laparotomic orthotopic tumor cell injection into the gastric submucosa	Laparotomic orthotopic tumor tissue inoculation beneath the gastric antrum	Percutaneous tumor cell injection into the peritoneum
Success rate	100% (12/12)	91.7% (11/12)	58.3% (7/12)
Major pathological events	Rapid tumor progression resulting in respiratory distress syndrome, diffused peritonitis due to perforation of gastric tumor, intestinal obstruction, renal failure	Rapid tumor progression resulting in intestinal obstruction, renal failure	Rapid tumor progression resulting in intestinal adhesion an obstruction, renal failure
Gross pathology	Ulcerative gastric cancer with PC, ascites	Ulcerative gastric cancer with PC, ascites	PC without gastric ulcer, ascites
Histopathology	Penetrating growth of cancer cell nests invading surrounding structures, tumor necrosis in the central zone of the tumor mass		
Advantages	Most resemble clinical gastric cancer with PC	Technically less difficult	Technically easy
Disadvantages	Technically difficult	Not exactly mimic gastric cancer with PC	Mistaken injection into the intestines

## Discussion

PC represents a serious clinical challenge in the treatment of gastrointestinal and gynecological cancers. In gastric cancer, PC is a frequent event with 15% to 50% or more patients having PC at the surgical exploration, especially when there is serosal involvement by the tumor [14-16]. Even after curative resection of gastric cancer, PC remains a major problem of postoperative recurrence. A Korean study in 500 gastric cancer patients treated by standardized radical gastrectomy and lymphadectomy, found that within 5 years post gastrectomy, PC is the most frequent pattern (51.7%) of cancer recurrence [17]. Another randomized prospective study in Japan also found peritoneal recurrence is the most frequent event (15.8%) at 3 years in 530 patients treated with highly standardized curative gastrectomy [18]. A prospective Italian study in 200 patients found that, at the mean follow-up of 42.3 months, PC accounted for 32.9% of recurrence [19]. Another Italian study with 441 gastric cancer patients showed 17% PC recurrence at the median follow-up of 48 months [20]. Therefore, synchronous and metachronous PC is the most important problem of gastric cancer recurrence and metastasis. Such gastric PC is associated with poor prognosis with median survival ranging from 1-1.6 months [21,22] to 3.1-9 months [1,15]. As is rightly stated, the risk of peritoneal recurrence of gastric cancer is particularly high in patients with diffuse-mixed tumors and infiltration of the serosa, against which surgery alone, no matter how radical, can offer little possibility of a cure [20]. Therefore, new comprehensive treatment strategies are required.

Clinical studies suggest that CRS plus HIPEC could achieve good efficacy in selected patients with PC. To our knowledge there are 4 institutional studies on CRS plus HIPEC in patients with gastric cancer, 2 retrospective (42 and 26 patients, respectively) [3,23], 1 prospective (49 patients) [4] and 1 comparative non-randomized (34 patients) studies [24]. The median survival ranged from 6.6 months [24], 8 months [23] to 10-11 months [3,4], and the 5-year survival ranged from 6% [3,25] to 16% [4]. These clinical studies are based on non-homogenous and non-standardized groups of patients. In order to more objectively evaluate such treatment, it is necessary to study this treatment modality under experimental conditions.

Small animal models of PC have been established, including nude mice models and rat models [26-29]. In most of these animal models, cancer cells are injected directly into the peritoneum, which will result in widespread PC in due time [25,30-32]. All these small animal models are only suitable for HIPEC alone because the small body size and limited blood supply cannot stand major surgical interventions. The establishment of large animal model of PC from gastric cancer is necessary for experimental studies testing CRS and HIPEC.

VX2 carcinoma is a rabbit tumor of epithelial origin, established from a virus-induced papilloma by Rous and coworkers [12]. Characterized by rapid tumor growth and early metastasis, this tumor is extremely malignant and can be allogeneously transplanted almost anywhere in rabbits. Although VX2 is a squamous cell carcinoma model, which may be different from adenocarcinoma, it has been used in many experimental studies on head and neck cancer [33-36], lung cancer [37], esophageal cancer [38,39], breast cancer [40], gastric cancer [41,42], liver cancer [43,44], colon cancer [45,46], kidney cancer [47-49], bladder cancer [50], bone tumor [51] and simple peritoneal carcinomatosis [52]. In this study we constructed a rabbit model of gastric cancer with PC. Our results demonstrated that the orthotopic inoculation of tumor cells into the stomach is the most appropriate method, resulting in typical ulcerative gastric cancer and progressive PC, all features similar to the clinico-pathologic progression of gastric cancer patients. In comparison, percutaneous injection of cancer cells into the abdominal cavity could result in intestinal injury by mistake. No ulcerative gastric cancer could be induced, and the tumor take rate is low. These results support the notion that orthotopic tumor model is preferred for the study of tumor biological behaviors and interventions [53]. The natural history of this model is about 4 weeks, including a subclinical stage in the first week, clinical stage in the second week, accelerated PC stage in the third week and terminal stage in the fourth week. Typical PC features are peritoneal cancer nodules of various sizes throughout the whole abdominal cavity, "omentum cake", intestinal obstruction and bloody ascites. The end of the first week could be considered as the early PC, while the end of the second week as the advanced PC. Therefore, specific treatment approaches could be designed to target either early PC or advanced PC.

## Conclusions

A rabbit model of gastric cancer with PC has been established by injecting VX2 cancer cells into the submucosal layer of the stomach. The model is characterized by typical ulcerative gastric cancer with progressive PC, making it more suitable for surgical interventional studies to evaluate CRS and HIPEC against gastric PC.

## Competing interests

The authors declare that they have no competing interests.

## Authors' contributions

LI Y conceived, designed and partly conducted the study. Mei LJ, Yang XJ, Tang L, and Hassan AHA conducted the study and drafted the manuscript. Yonemura Y gave technique instructions and revised the manuscript. All authors have read the approved the final manuscript.

## Pre-publication history

The pre-publication history for this paper can be accessed here:

http://www.biomedcentral.com/1471-2407/10/124/prepub
